# Identification and isolation of insecticidal oxazoles from *Pseudomonas* spp.

**DOI:** 10.3762/bjoc.8.85

**Published:** 2012-05-18

**Authors:** Florian Grundmann, Veronika Dill, Andrea Dowling, Aunchalee Thanwisai, Edna Bode, Narisara Chantratita, Richard ffrench-Constant, Helge B Bode

**Affiliations:** 1 Department of Molecular Biotechnology, Goethe-Universität Frankfurt, Max-von-Laue-Str. 9, 60438 Frankfurt am Main, Germany; 2 Biosciences, University of Exeter, Penryn, Cornwall, TR10 9EZ, UK; 3 Faculty of Tropical Medicine, Mahidol University, 420/6 Ratchawithi Road, Ratchathewi, Bangkok 10400, Thailand

**Keywords:** insecticidal activity, labradorin, oxazole, *Pseudomonas*, secondary metabolite

## Abstract

Two new and five known oxazoles were identified from two different *Pseudomonas* strains in addition to the known pyrones pseudopyronine A and B. Labeling experiments confirmed their structures and gave initial evidence for a novel biosynthesis pathway of these natural oxazoles. In order to confirm their structure, they were synthesized, which also allowed tests of their bioactivity. Additionally, the bioactivities of the synthesis intermediates were also investigated revealing interesting biological activities for several compounds despite their overall simple structures.

## Findings

**Figure 1:**
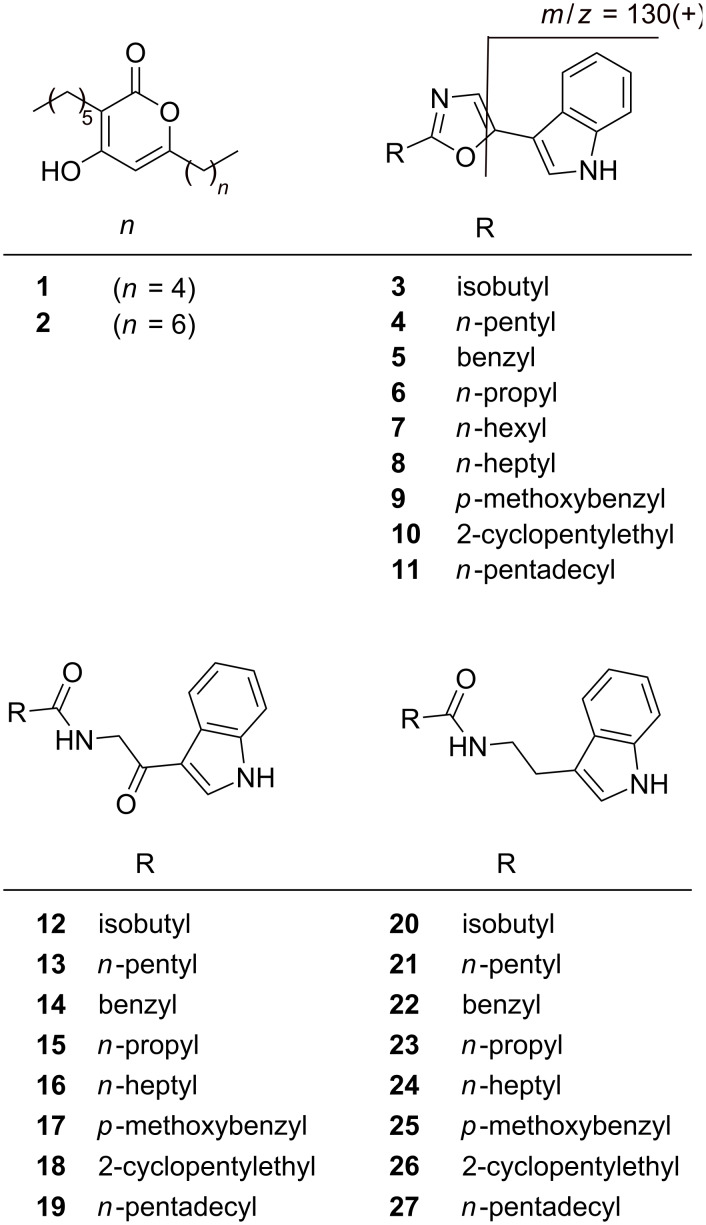
Structures of pseudopyronines A (**1**) and B (**2**) and natural oxazoles (**3**–**8**) as well as synthetic oxazole derivatives (**9**–**11**). All natural and synthetic oxazoles show the characteristic MS fragment 130 *m*/*z* [M + H]^+^. The intermediates of the oxazole syntheses (**12**–**27**) are also shown as they were also tested for their biological activity.

During our search for novel natural products from entomopathogenic bacteria, strain PB22.5 was isolated from a soil sample collected in Thailand by using the baiting technique for the isolation of entomopathogenic bacteria and/or entomopathogenic nematodes, as described previously [1–4]. HPLC–MS analysis of extracts from strain PB22.5 grown in LB showed several peaks (Supporting Information File 1, Figure S1) that have not been detected in other entomopathogenic bacteria (data not shown). Subsequent large scale cultivation and purification of the main components led to the isolation of five major compounds, which were subjected to structure elucidation by MS (Supporting Information File 1, Figure S2) and NMR analysis (Supporting Information File 1, Tables S1–S6). Whereas the structures **1** and **2** could be identified as pseudopyronines A (**1**, 267.4 *m*/*z* [M + H]^+^, C_16_H_26_O_3_) and B (**2**, 295.4 *m*/*z* [M + H]^+^, C_18_H_30_O_3_) [5–7], three other compounds were identified as the known oxazole derivatives labradorin 1 (**3**, 241.1 *m*/*z* [M + H]^+^, C_15_H_16_N_2_O), labradorin 2 (**4**, 255.2 *m*/*z* [M + H]^+^, C_16_H_18_N_2_O) and pimprinaphine (**5**, 275.1 *m*/*z* [M + H]^+^, C_18_H_14_N_2_O) (Figure 1) [8–9].

Detailed analysis of the HPLC–MS data showed WS-30581 A (**6**, 227.1 *m*/*z* [M + H]^+^, C_14_H_14_N_2_O) [10] as an additional oxazole derivative, but which was only produced in minute amounts. WS-30581 A (**6**) was identified by comparing the retention time and the MS fragmentation (Supporting Information File 1, Figure S2) of the product contained in the extract, with a synthesized compound. All oxazoles showed a characteristic fragment ion of 130 *m*/*z* [M + H]^+^ (Supporting Information File 1, Figure S2), and labeling experiments (Supporting Information File 1, Figure S3) enabled the elucidation of this ion as 3-methylidene-3*H*-indolium (Figure 1).

In order to determine the genus of the producing strain PB22.5, we sequenced its 16S-rRNA gene revealing it to be a *Pseudomonas* sp. with closest homology to *P. putida* (100% similarity: Supporting Information File 1, Figure S4) [11–14].

As judged on the basis of high-resolution MALDI–MS and LC–ESIMS/MS data the well-known entomopathogenic *P. entomophila* [15–16] also produces compounds **3**–**6** but not **1** and **2**. Furthermore two novel oxazole derivatives named labradorin 3 (**7**, 268.4 *m*/*z* [M + H]^+^, C_17_H_20_N_2_O) and labradorin 4 (**8**, 283.2 *m*/*z* [M + H]^+^, C_18_H_22_N_2_O) were detected, but they could not be isolated due to their very low production.

**Figure 2:**
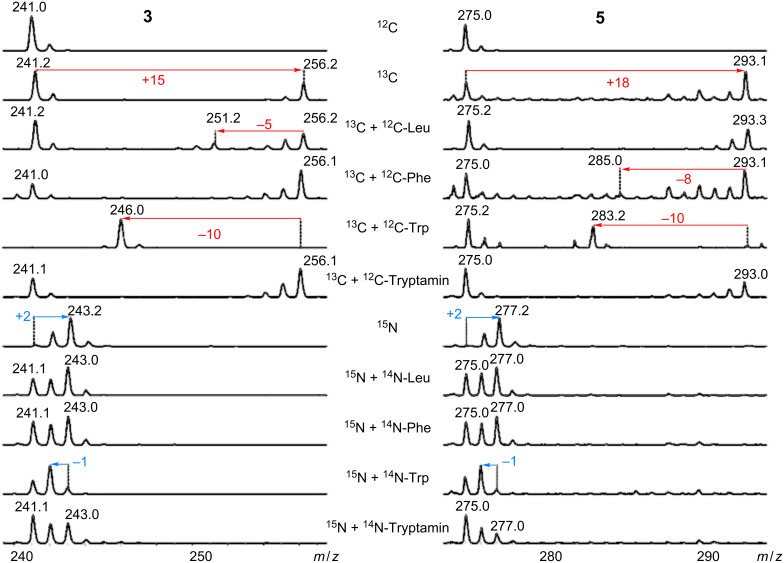
MS data from strain PB22.5, which was cultivated in [U-^13^C] and [U-^15^N] medium background and LB medium as a control. Feeding experiments with ^12^C and ^14^N amino acids confirmed structures **3** and **5** and gave initial insights into the biosynthesis.

Labeling experiments were performed in order to confirm the oxazole structures and to reveal their biosynthesis. Therefore, both strains were cultivated in fully labeled ^13^C or ^15^N media and ^12^C precursors were added (Figure 2).

**Figure 3:**
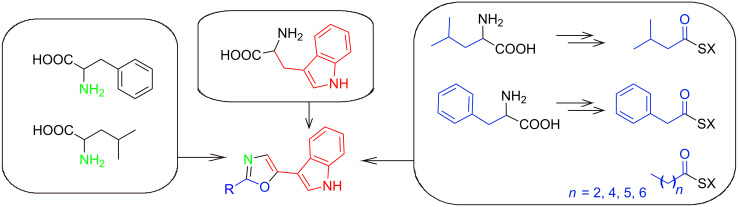
All incorporated biosynthetic precursors of the oxazoles are shown in color. The nitrogen shown in green is derived from transamination as part of the amino acid metabolism. SX = activated ester, which may be coenzyme A or enzyme (acyl carrier protein or polyketide synthase or nonribosomal peptide synthetase) bound.

Five carbons of leucine are incorporated in compound **3**, while eight carbons of compound **5** originate from phenylalanine**,** which confirms these moieties to be amino-acid derived (Figure 3).

Ten carbons and one nitrogen of tryptophan were incorporated in all oxazoles, confirming the indole moiety to be derived from tryptophan. Surprisingly, no incorporation of carbon from tryptamine was observed, and the fact that nitrogen labeling also occurs when ^14^N-tryptamine, leucine or phenylalanine is fed indicates an efficient transaminase activity in both strains, as one would indeed expect for a bacterial strain living on proteinogenic substrates such as insects. Indole acetaldehyde was also fed in ^13^C-labeled medium, but, in this case as well, no incorporation was observed (data not shown).

Thus, indolepyruvate or an unknown degradation product thereof is proposed as a precursor for the indole moiety. Unfortunately, the production of **7** and **8** was insufficient for analysis in the labeled media, but the nature of the side chains was concluded from a fatty-acid analysis of the producing strain, which showed only straight-chain fatty acids (data not shown).

In order to confirm the structure of all oxazole derivatives, to compare their retention times, and to provide enough material for bioactivity tests, oxazoles **3**–**6** and **8** were synthesized. Briefly, the respective tryptamine derivatives were formed, which were then oxidized at the alpha-position with the help of 2,3-dichloro-5,6-dicyano-1,4-benzoquinone (DDQ) and cyclized to give the oxazoles by using phosphorylchloride as described [9]. Additionally, three nonnatural derivatives were synthesized to get a more diverse set of oxazole derivatives differing from the natural compounds by unusual (compound **9** and **10**) or very long (compound **11**) acyl chains.

Additionally, the synthetic intermediates were also tested as precursors in labeling experiments as described above, but again no incorporation could be observed. Thus, the biosynthesis via tryptamine amides, which are subsequently oxidized and cyclized to give the oxazoles, can be excluded and more experiments are needed to fully elucidate the biosynthesis of these simple heterocyclic compounds.

All synthesized compounds and intermediates thereof were tested for their biological activity, as a broad range of activities has already been described for these oxazoles, including anticonvulsant and antithrombotic activity as well as activity against human lung and pancreas cancer cell lines [9–10,17].

We tested the bioactivity against the human breast-cancer cell line MCF-7, using the XTT assay [18], and against insect hemocytes from the greater wax moth *Galleria melonella* according to the method described by Proschak et al. [19]. Three oxazole compounds show LD_50_ [µg ml^−1^] values against *Galleria* hemocytes of 30 (compound **3**), 1.7 (compound **6**), and 103 (compound **9**) (Table S7). Compounds **3**, **5**, **6**, and **9** are active in the MCF-7 assay with EC_50_ [µg ml^−1^] values of 58, 363, 26, and 34, respectively. Similar to previous results [19], several tryptamine amide derivatives (**20**–**24**, **26**) showed cytotoxic activity against the MCF-7 cells with **24** being the most potent compound (1.02 µg ml^−1^). Interestingly, **26** also showed activity against *Galleria* hemocytes, although its oxazole derivative **10** did not, which may point to different targets for both compound classes.

The class of oxazole compounds, which were identified in this article, are not only prevalent in *Pseudomonas* but also in other bacteria such as *Streptomyces* [20] or *Streptoverticillium* [8,10], suggesting a biological relevance also in these bacteria. However, as concluded from the observed activity against insect cells, they could significantly add to the overall insecticidal activity of the investigated *Pseudomonas* strains.

## Supporting Information

File 1 General experimental procedures, isolation of the strain and taxonomic identification, cultivation and extraction, isolation, labeling experiments, synthesis, bioactivity results and compound characterization.

## References

[1] Akhurst R J (1980). J Gen Microbiol.

[2] Bedding R A, Akhurst R J (1975). Nematologica.

[3] White G F (1927). Science.

[4] Woodring L J H, Kaya K H (1988). Steinernematid and Heterorhabditid nematodes. A Handbook of biology and techniques.

[5] Chu M, Mierzwa R, Xu L, He L, Terracciano J, Patel M, Zhao W, Black T A, Chan T M (2002). J Antibiot.

[6] Kong F, Singh M P, Carter G T (2005). J Nat Prod.

[7] Singh M P, Kong F M, Janso J E, Arias D A, Suarez P A, Bernan V S, Petersen P J, Weiss W J, Carter G, Greenstein M (2003). J Antibiot.

[8] Koyama Y, Yokose K, Dolby L J J (1981). Agric Biol Chem.

[9] Pettit G R, Knight J C, Herald D L, Davenport R, Pettit R K, Tucker B E, Schmidt J M (2002). J Nat Prod.

[10] Umehara K, Yoshida K, Okamoto M, Iwami M, Tanaka H, Kohsaka M, Imanaka H (1984). J Antibiot.

[11] Kimura M (1980). J Mol Evol.

[12] Saitou N, Nei M (1987). Mol Biol Evol.

[13] Tamura K, Dudley J, Nei M, Kumar S (2007). Mol Biol Evol.

[14] Thompson J D, Higgins D G, Gibson T J (1994). Nucleic Acids Res.

[15] Vodovar N, Vinals M, Liehl P, Basset A, Degrouard J, Spellman P, Boccard F, Lemaitre B (2005). Proc Natl Acad Sci U S A.

[16] Vodovar N, Vallenet D, Cruveiller S, Rouy Z, Barbe V, Acosta C, Cattolico L, Jubin C, Lajus A, Segurens B (2006). Nat Biotechnol.

[17] Naik S R, Harindran J, Varde A B (2001). J Biotechnol.

[18] Scudiero D A, Shoemaker R H, Paull K D, Monks A, Tierney S, Nofziger T H, Currens M J, Seniff D, Boyd M R (1988). Cancer Res.

[19] Proschak A, Schultz K, Herrmann J, Dowling A J, Brachmann A O, ffrench-Constant R, Müller R, Bode H B (2011). ChemBioChem.

[20] Joshi B S, Taylor W I, Bhate D S, Karmarkar S S (1963). Tetrahedron.

