# Supplementary material for: Identification and isolation of insecticidal oxazoles from Pseudomonas spp
Source: Beilstein J Org Chem. 2012 May 18;8:749–52. doi: 10.3762/bjoc.8.85 (PMC3388863; doi:10.3762/bjoc.8.85)
Supplement: File 1 — General experimental procedures, isolation of the strain and taxonomic identification, cultivation and extraction, isolation, labeling experiments, synthesis, bioactivity results and compound characterization. [file Beilstein_J_Org_Chem-08-749-s001.pdf]

Supporting Information

for

Identification and isolation of insecticidal oxazoles from *Pseudomonas* sp.

Florian Grundmann[1], Veronika Dill[1], Andrea Dowling [2], Aunchalee Thanwisai[3], Edna Bode[1], Narisara Chantratita[3], Richard ffrench-Constant [2], and Helge B. Bode[1]*

Address: [1]Department of Molecular Biotechnology, Goethe-Universität Frankfurt, Max-von-Laue-Str. 9, 60438 Frankfurt am Main, Germany, [2]Biosciences, University of Exeter, Penryn, Cornwall, TR10 9EZ UK and [3]Faculty of Tropical Medicine, Mahidol University, 420/6 Ratchawithi Road, Ratchathewi, Bangkok 10400, Thailand

Email: Helge B. Bode - h.bode@bio.uni-frankfurt.de

* Corresponding author

General experimental procedures, isolation of the strain and taxonomic identification, cultivation and extraction, isolation, labeling experiments, synthesis, bioactivity results and compound characterization.

Content

1) General experimental procedures S3
2) Isolation of the strain and taxonomic identification S4
3a) Cultivation and extraction S5
3b) Isolation S6
4) Feeding experiments S6
5) Synthesis S7
6) Compound characterization S8
Table S1: ^{1}H NMR of compounds **3–11**. S18
Table S2: ^{13}C NMR of compounds **3–11**. S19
Table S3: ^{1}H NMR of compounds **12–19**. S20
Table S4: ^{13}C NMR of compounds **12–19**. S21
Table S5: ^{1}H NMR of compounds **20–27**. S22
Table S6: ^{13}C NMR of compounds **20–27**. S23
Table S7: Bioactivity results (- = not active; * = crystallization at concentrations required for the assay). Compounds **12–19** were not active in the MCF-7 cell assay and crystallized at concentrations required for the *Galleria* assay. S24
Figure S1: A) HPLC–MS base-peak chromatograms of the crude extract and extracted ion chromatograms of the natural compounds of *P.entomophila* (**A**) and PB22.5 (**B**). The different moieties of the oxazoles are shown above the chromatogram. S25
Figure S2: MS/MS spectra of the synthetic products **3–8** and the identified natural oxazoles **7** (g) and **8** (f) from *P. entomophila.* The characteristic oxazole fragment is shown in red. S26
Figure S3: MS/MS spectra of compound **3** with isotopic labeling as proof of the structure of the characteristic fragment *m*/*z* = 130. .... S27
Figure S4: Phylogenetic relationships of PB22.5 and *Pseudomonas* sp. The tree was constructed by using the partial 16S rDNA gene sequence (537 bp), the model of Kimura 2-parameter and the Neighbor-Joining module of MEGA software version 4.0.2. A bootstrap value is 1,000 replicates. The bar indicates 0.5% sequence divergence. Numbers after each species indicate the GenBank Accession numbers. S27
References S28

1) General experimental procedures

Solvents and reagents were obtained from Sigma–Aldrich (München, Germany) or Acros (Geel, Belgium). The UPLC–ESIMS analysis was performed on a Ultimate 3000 system (Dionex, Idstein, Germany) by using a RP C18 BEH Acquity UPLC 50 mm × 2.1 mm × 1.7 µm (Waters, USA) as column, coupled with an Amazon X ion trap MS (Bruker, Bremen, Germany). All chromatography was performed with a binary mixture of water and acetonitrile with 0.1% formic acid (gradient: 0–22 min, 5–95% acetonitrile, MS: positive and negative alternating polarity mod, scan range: 100–1200 *m*/*z,* target mass: 600 *m*/*z*, MS 2 Frag Ampl. 1.00 V). The data was processed with the DataAnalysis Software version 4.0 SP2 (Bruker, Bremen, Germany). The general gradient, which was used for identification and characterization of all compounds, was with a flow of 0.6 mL/min from 0–2 min 5%, 2–14 min 5–95%, 14–17 min 95% acetonitrile. Silica chromatography was performed on a Biotage SP1™ Flash Purification System (Biotage, Uppsala, Sweden) using 40+M Cartridges KP-Sil 40 × 150 mm (Biotage, Uppsala, Sweden) with an UV detector. UV spectra were recorded on a Jasko Spectrophotometer 650 (Essex, UK) and IR spectra measured on a Jasco FT/IR-420 spectrometer. ^{1}H, COSY, HSQC, HMBC NMR spectra were recorded on a Bruker AV 400 MHz spectrometer and ^{13}C data were acquired on a Bruker AV 300 MHz spectrometer (Bruker, Billerica, USA). Chemical shifts δ are given in ppm referring to the signal center using the solvent peaks for reference: $CDCl_3$ 7.26 ppm/77.0 ppm and DMSO-d_6 2.49 ppm/39.7 ppm. HR-MALDI-MS data were analyzed with a MALDI LTQ Orbitrap XL (Thermo Fisher Scientific, Inc., Waltham, MA) equipped with a nitrogen laser at 337 nm and used in the Fourier transformation mode. The used instrument parameters were: laser energy, 16 µJ; automatic gain control, on; auto spectrum filter, off; scan mode, full; resolution, 100000; plate motion, survey CSPS. Spectra were analyzed and

monoisotopic mass lists were generated by using Qual Browser (version 2.0.7; Thermo Fisher Scientific, Inc., Waltham, MA). This software was also used for the generation of elemental formulas from highly accurate masses. The following adjustments were used for identification: charge, 1; nitrogen rule, do not use; mass tolerance, 3 ppm; RDB equiv, 0–100; possible elements: C, H, N, and O, respectively.

2) Isolation of the strain and taxonomic identification

The soil sample PB22.5 was collected from Tha Phon sub district, Muang district, Petchaboon province, Thailand. GPS location was 16° 32' 34.6" N and 101° 08' 47.6" E. The altitude was 143 m. Entomopathogenic nematode was recovered from soil samples by the insect-baiting technique [1]. Briefly, 500 g of a soil sample was put in a plastic container. Five last instar larvae of *G. mellonella* were placed on top of the soil and the container was covered with a lid. The box was turned upside down to let the *G. mellonella* move in the soil from the bottom and stored at 20–25 °C. After five days, dead *G. mellonella* were collected. Individual *G. mellonella* cadaver were placed in white traps [2] and held at 20–25 °C to allow for the emergence of the infective juvenile nematodes (IJs). Emerging nematodes were pooled for each sample and used to infect fresh *G. mellonella* larvae to confirm pathogenicity and to produce nematodes used for identification and establishment of nematode cultures. The nematodes were kept in aerated water and stored at 13 °C. Bacteria were isolated from the haemolymph of insect cadavers and cultured on nutrient bromothymol blue agar (NBTA) supplemented with 0.004% (w/v), triphenyltetrazolium chloride (TTC) and 0.0025% (w/v) bromothymol blue [1,3] at rt for 4 d. Sequencing of the 16S rRNA gene was performed following genomic DNA isolation using standard

protocols. The resulting sequences were compared to sequences of 16S rRNA of *Pseudomonas sp.*, which are available in GenBank. Multiple sequences were aligned by using Clustal W [1,4] included in the MEGA version 5.05 software [5]. Phylogenetic distance trees were calculated by using the Kimura two-parameter model and the Neighbor-Joining module [6] of Molecular Evolutionary Genetics Analysis (MEGA) software version 4.0.2. Bootstrap analysis was carried out with 1,000 datasets. Strain PB22.5 was identified as *Pseudomonas putida* with 100% similarity (Figure S1).

3a) Cultivation and extraction

Strain PB22.5 was maintained on LB-agar (1.5% w/v agar) and grown at 30 °C for 72 h. For the investigation of the secondary metabolite production by HPLC–MS/MS, 20 mL LB-medium (5 g NaCl, 5 g yeast extract, 10 g Trypton in 1 L water) with 2% Amberlite XAD 16 adsorber resin (Sigma–Aldrich, München, Germany) in a 50 mL flask was inoculated with a single colony from solid medium and cultivated for 72 h under permanent shaking at 200 rpm. The XAD beads were collected by sieving and then extracted with 20 mL methanol. After evaporation of the methanol the obtained crude brown oil was mixed with 2 mL methanol and centrifuged at 13300 rpm for 15 min. Then the debris-free supernatant was diluted 1:1 with methanol. For the isolation of the secondary metabolites three precultures with 20 mL LB medium (pH 7.5) each in 50 mL flasks were inoculated with a single colony from solid medium and cultivated for 17 h at 30 °C and 180 rpm. Subsequently, 10 mL of the preculture was used to inoculate 5 × 5 L flasks containing 1 L of LB with 2% XAD and the cultures were shaken at 130 rpm for 72 h at 30 °C.

3b) Isolation

The XAD beads were collected by sieving and extracted three times with 1000 mL methanol (30 min of stirring). After evaporation, the obtained crude brown oil (5.09 g) was separated by flash chromatography by using a quarternary gradient of hexane, ethylacetate, chloroform and methanol (flow rate: 40 ml/min, 40+M Cartridges KP-Sil 40 × 150 mm). The obtained fractions were analyzed by TLC and UPLC–MS and combined according to their similar content. The fraction containing **3**–**5** (110 mg) was further separated on a preparative HPLC system with a binary gradient of water and acetonitrile. The gradient was from 0–10 min 40% acetonitrile and from 10–21 min 40–70% acetonitrile. Preparative HPLC was carried out on a Waters Autopurification system coupled with a 3100 Mass Detector using an XBridge™ C18 5 µm 4.6 × 150 mm column (Waters, USA) for analytical scale, and an XBridge™ C18 5 µm OBD™ 19 × 150 mm column for preparative scale. The data was processed with the MassLynx Software version 627 (Waters, USA). Whereas **4** was isolated (2 mg, R_t = 18.3 min) in a pure form, **3** and **5** were isolated together in one fraction, which was separated into the two single compounds through an additional flash purification step with a binary gradient of hexane and ethylacetate.

4) Feeding experiments

The incorporation of amino acids was tested by feeding L-tryptophan, L-leucine, and L-phenylalanine to strain PB22.5. For the elucidation of the biosynthesis, all synthesized intermediates and indole acetaldehyde were also fed. Cultures were cultivated in [U-^{13}C] (ISOGRO® C-powder growth medium 99 atom% C, Sigma–Aldrich) and [U-^{15}N] medium (ISOGRO® N-powder growth medium 99 atom% N, Sigma–Aldrich) background. The medium also contained 10 mM K_2HPO_4, 10 mM KH_2PO_4, 8 mM $MgSO_4 \cdot 7\ H_2O$, and 90 µM $CaCl_2 \cdot H_2O$. Strain PB22.5 was cultivated

overnight at 30 °C in 20 mL LB medium. After centrifugation at 11000 rpm for 1 min, the supernatant was removed. The cell pellet was resuspended in ^{13}C-medium and ^{15}N-medium, respectively. This washing step was carried out three times. Then, 400 µL of the washed cells was used to inoculate 4.6 mL of medium. After 2 h the amino acids (biochemical grade, Roth, Karlsruhe) or other precursors were fed in portions of 1 mM every 12 h.

5) Synthesis

All compounds were synthesized, with little modifications, as described [7] starting with 4 mmol of tryptamine as shown below.

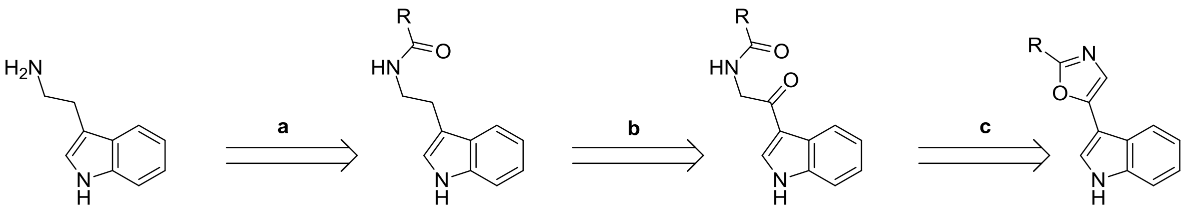

General Synthesis **a**: The tryptamine was dissolved in tetrahydrofurane (THF) under nitrogen. Two equiv of hexamethylsilazane and 1.2 equiv of the corresponding acid chloride were added dropwise and the solution was stirred at rt overnight. The work-up started with an acidification with 1 M HCl. After dilution with water, the organic phase was extracted three times with ethylacetate, washed with 1 M NaOH and saturated NaCl solution. The combined fractions were dried over Na_2SO_4, concentrated and purified by flash chromatography (hexane/ethylacetate 1:1) to yield the product as a white solid (except **26**).

General synthesis **b**: The educt was dissolved in a 9:1 mixture of THF and water, and then 2 equiv of DDQ were added. After stirring for 2 h at rt the reaction mixture was concentrated, diluted with 0.1 M NaOH and mixed vigorously. The resulting

suspension was filtered and the filter cake was washed with cold ethyl acetate to yield the product as a white solid.

General synthesis **c**: The educt was dissolved in phosphorus oxychloride (40 equiv) under nitrogen and heated under reflux overnight. The cooled mixture was poured into a mixture of methanol and ice water (1:4) and extracted three times with dichloromethane. The organic layer was washed with saturated NaCl solution and evaporated. The crude product was purified by flash chromatography (hexane/ethylacetate 4:1) to yield the product as a white solid.

6) Compound characterization

All compounds were characterized by ^{1}H and ^{13}C NMR spectroscopy, HRMS and ESI–MS/MS. All new compounds were characterized by UV, IR as well as 1D and 2D NMR spectroscopy. ^{1}H/^{1}H-COSY correlations (bold lines) and the most important HMBC correlations (arrows) are shown in the structures of the new compounds.

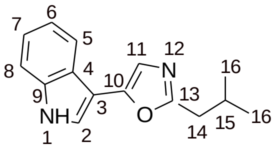

3

Compound **3**, labradorin 1: white solid; ^{1}H NMR ($CDCl_3$) see Table S1; ^{13}C NMR ($CDCl_3$) see Table S2; HPLC–MS (general gradient) R_t = 9.1 min; MS^2 212.0, 198.9, 184.0, 170.9, 157.0, 130.0, 122.0, 105.0; (+)-HRMS 241.1335 *m/z* $[M + H]^+$ (calcd for $C_{15}H_{16}N_2O$ *m/z* $[M + H]^+$ 241.1335).

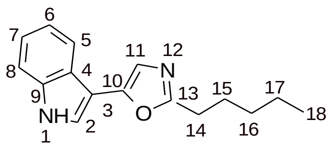

4

Compound **4**, labradorin 2: white solid; ^{1}H NMR ($CDCl_3$) see Table S1; ^{13}C NMR ($CDCl_3$) see Table S2; HPLC–MS (general gradient) R_t = 9.9 min; MS^2 237.1, 227.1, 210.0, 199.9, 183.0, 168.0, 157.0, 142.0, 130.0; (+)-HRMS 255.1493 *m*/*z* $[M + H]^+$ (calcd for $C_{16}H_{18}N_2O$ *m*/*z* $[M + H]^+$ 255.1492).

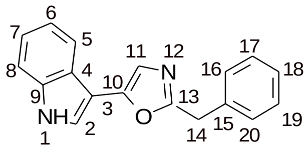

5

Compound **5**, pimprinaphine: white solid; ^{1}H NMR ($CDCl_3$) see Table S1; ^{13}C NMR ($CDCl_3$) see Table S2; HPLC–MS (general gradient) R_t = 9.0 min; MS^2 257.9, 247.0, 230.0, 217.9, 196.9, 182.0, 157.9, 142.0, 130.0; (+)-HRMS 275.1178 *m*/*z* $[M + H]^+$ (calcd for $C_{18}H_{14}N_2O$ *m*/*z* $[M + H]^+$ 275.1179).

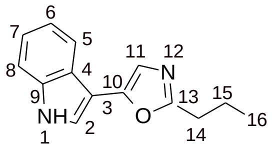

6

Compound **6**, WS-30582: white solid; ^{1}H NMR ($CDCl_3$) see Table S1; ^{13}C NMR ($CDCl_3$) see Table S2; HPLC–MS (general gradient) R_t = 8.5 min; MS^2 208.8, 200.8, 197.9, 184.9, 182.0, 169.9, 157.0, 142.0, 130.0; (+)-HRMS 227.1183 *m*/*z* $[M + H]^+$ (calcd for $C_{14}H_{14}N_2O$ *m*/*z* $[M + H]^+$ 227.1179).

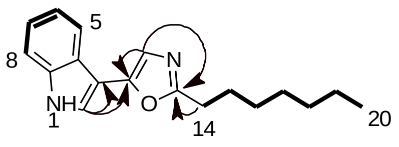

8

Compound **8**, labradorin 4: white solid; UV ($CHCl_3$) λ_{max} (ε) 263.0 (11000), 239.0 (11000); IR ν_{max}/cm^{-1} 3431, 3132, 2924, 2852, 1637, 1570, 1454, 1388, 1251, 1182, 1124, 1112; ^{1}H NMR ($CDCl_3$) see Table S1; ^{13}C NMR ($CDCl_3$) see Table S2; HPLC–MS (general gradient) R_t = 11.2 min; MS^2 255.1, 240.0, 228.1, 197.9, 184.0, 157.0, 138.1, 130.0; (+)-HRMS 283.1806 *m*/*z* $[M + H]^+$ (calcd for $C_{18}H_{22}N_2O$ *m*/*z* $[M + H]^+$ 283.1805).

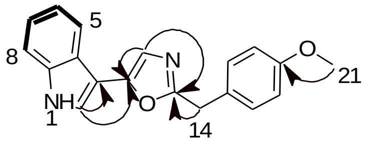

9

Compound **9**, 3-[2-(4-methoxybenzyl)-1,3-oxazol-5-yl]-1*H*-indole: white solid; UV ($CDCl_3$) λ_{max} (ε) 274.0 (11100), 238.5 (12500); IR ν_{max}/cm^{-1} 3419, 3168, 2929, 2854, 1637, 1611, 1572, 1511, 1455, 1247, 1116, 1033; ^{1}H NMR ($CDCl_3$) see Table S1; ^{13}C NMR ($CDCl_3$) see Table S2; HPLC–MS (general gradient) R_t = 9.1 min; MS^2 196.9, 142.0, 131.0; (+)-HRMS 305.1284 *m*/*z* $[M + H]^+$ (calcd for $C_{19}H_{16}N_2O_2$ *m*/*z* $[M + H]^+$ 305.1285).

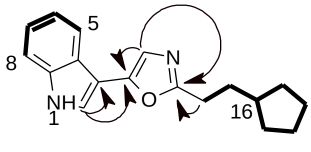

10

Compound **10**, 3-[2-(2-cyclopentylethyl)-1,3-oxazol-5-yl]-1*H*-indole: white solid; UV ($CHCl_3$) λ_{max} (ε) 264.0 (8700), 238.5 (11000); IR ν_{max}/cm^{-1} 3413, 3145, 2927, 2864, 1628, 1609, 1568, 1446, 1240, 1117; ^{1}H NMR ($CDCl_3$) see Table S1; ^{13}C NMR ($CDCl_3$) see Table S2; HPLC–MS (general gradient) R_t = 10.6 min; MS^2 253.9, 239.0, 199.0, 185.0, 171.0, 159.0, 130.0, 108.0; (+)-HRMS 281.1658 *m*/*z* [M + H]$^+$ (calcd for $C_{18}H_{20}N_2O$ *m*/*z* [M + H]$^+$ 281.1648).

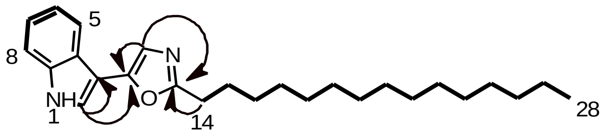

11

Compound **11**, 3-(2-pentadecyl-1,3-oxazol-5-yl)-1*H*-indole: white solid; UV ($CHCl_3$) λ_{max} (ε) 262.0 (11000), 239.0 (11500); IR ν_{max}/cm^{-1} 3421, 3171, 2922, 2849, 1634, 1569, 1452, 1116; ^{1}H NMR ($CDCl_3$) see Table S1; ^{13}C NMR ($CDCl_3$) see Table S2; HPLC–MS (general gradient) R_t = 15.4 min; MS^2 377.0, 359.0, 335.6, 329.2, 308.1, 294.7, 290.2, 247.7, 240.7, 225.0, 197.9, 165.9, 159.0, 157.0, 130.1; (+)-HRMS 395.3056 *m*/*z* [M + H]$^+$ (calcd for $C_{26}H_{38}N_2O$ *m*/*z* [M + H]$^+$ 395.3057).

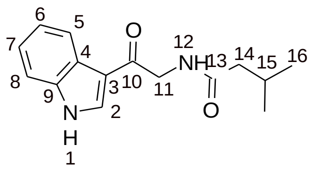

12

Compound **12**, *N*-[2-(1*H*-indol-3-yl)-2-oxoethyl]-3-methylbutanamide: white solid; ^{1}H NMR (DMSO-d_6) see Table S3; ^{13}C NMR (DMSO-d_6) see Table S4; HPLC–MS (general gradient) R_t = 7.1 min; MS^2 241.0, 175.0, 142.0, 114.0; (+)-HRMS 259.1444 *m*/*z* [M + H]$^+$ (calcd for $C_{15}H_{18}N_2O_2$ *m*/*z* [M + H]$^+$ 259.1441).

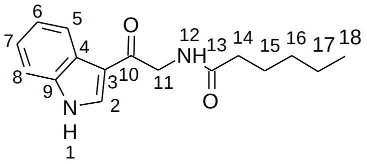

13

Compound **13**, *N*-[2-(1*H*-indol-3-yl)-2-oxoethyl]hexanamide: white solid; ^{1}H NMR (DMSO-d_6) see Table S4; ^{13}C NMR (DMSO-d_6) see Table S4; HPLC–MS (general gradient) R_t = 8.0 min; MS^2 255.0, 175.0, 156.0, 128.1; (+)-HRMS 273.1605 *m*/*z* $[M + H]^+$ (calcd for $C_{16}H_{22}N_2O_2$ *m*/*z* $[M + H]^+$ 273.1598).

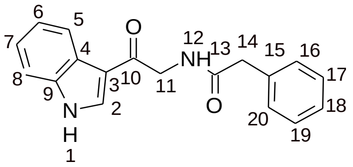

14

Compound **14**, *N*-[2-(1*H*-indol-3-yl)-2-oxoethyl]-2-phenylacetamide: white solid; ^{1}H NMR (DMSO-d_6) see Table S3; ^{13}C NMR (DMSO-d_6) see Table S4; HPLC–MS (general gradient) R_t = 7.7 min; MS^2 176.0, 148.1, 120.1; (+)-HRMS 293.1294 *m*/*z* $[M + H]^+$ (calcd for $C_{18}H_{16}N_2O_2$ *m*/*z* $[M + H]^+$ 293.1285).

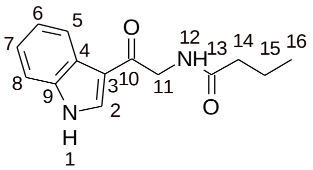

15

Compound **15**, *N*-[2-(1*H*-indol-3-yl)-2-oxoethyl]butanamide: white solid; ^{1}H NMR (DMSO-d_6) see Table S3; ^{13}C NMR (DMSO-d_6) see Table S4; HPLC–MS (general

gradient) R_t = 6.5 min; MS^2 227.0, 175.0, 128.0, 100.1; (+)-HRMS 245.1293 *m*/*z* [M + H]$^+$ (calcd for $C_{14}H_{18}N_2O_2$ *m*/*z* [M + H]$^+$ 245.1285).

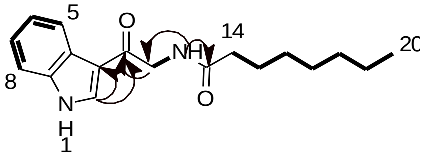

16

Compound **16**, *N*-[2-(1*H*-indol-3-yl)-2-oxoethyl]octanamide: white solid; UV (DMSO) λ_{max} (ε) 295.0 (11000), 258.0 (8800); IR ν_{max}/cm^{-1} 3328, 3189, 2953, 2923, 2854, 1655, 1626, 1542, 1517, 1457, 1435, 1239, 1157; ^{1}H NMR (DMSO-d_6) see Table S3; ^{13}C NMR (DMSO-d_6) see Table S4; HPLC–MS (general gradient) R_t = 9.4 min; MS^2 283.0, 184.0, 175.0, 156.0, 127.0; (+)-HRMS 301.1914 *m*/*z* [M + H]$^+$ (calcd for $C_{18}H_{24}N_2O_2$ *m*/*z* [M + H]$^+$ 301.1911).

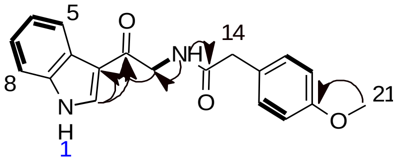

17

Compound **17**, *N*-[2-(1*H*-indol-3-yl)-2-oxoethyl]-2-(4-methoxyphenyl)acetamide: white solid; UV (DMSO) λ_{max} (ε) 295.0 (12200); IR ν_{max}/cm^{-1} 3412, 3294, 3184, 1654, 1617, 1539, 1510, 1437, 1247; ^{1}H NMR (DMSO-d_6) see Table S3; ^{13}C NMR (DMSO-d_6) see Table S4; HPLC–MS (general gradient) R_t = 7.7 min; MS^2 305.0, 206.0, 178.0, 150.1; (+)-HRMS 323.1395 *m*/*z* [M + H]$^+$ (calcd for $C_{19}H_{18}N_2O_3$ *m*/*z* [M + H]$^+$ 323.1390).

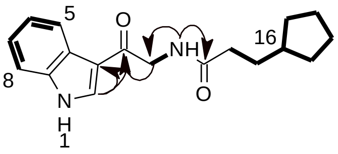

18

Compound **18**, 3-cyclopentyl-*N*-[2-(1*H*-indol-3-yl)-2-oxoethyl]propanamide: white solid; UV (DMSO) λ_{max} (ε) 295.0 (8400), 259.0 (10500); IR ν_{max}/cm^{-1} 3316, 3188, 2944, 2854, 1652, 1626, 1542, 1516, 1435, 1312, 1239, 1159; ^{1}H NMR (DMSO-d_6) see Table S3; ^{13}C NMR (DMSO-d_6) see Table S4; HPLC–MS (general gradient) R_t = 8.7 min; MS^2 281.0, 182.0, 175.0, 154.0; (+)-HRMS 299.1758 *m/z* [M + H]$^+$ (calcd for $C_{18}H_{22}N_2O_2$ *m/z* [M + H]$^+$ 299.1754).

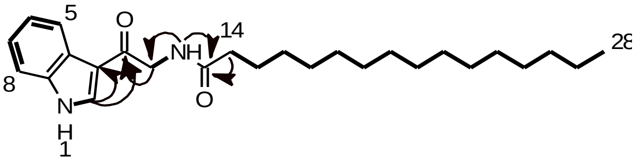

19

Compound **19**, *N*-[2-(1*H*-indol-3-yl)-2-oxoethyl]hexadecanamide: white solid; UV (DMSO) λ_{max} (ε) 295.0 (11000), 258.5 (8800); IR ν_{max}/cm^{-1} 3330, 3189, 2918, 2850, 1656, 1627, 1543, 1516, 1436, 1311, 1239, 1156; ^{1}H NMR (DMSO-d_6/$CDCl_3$) see Table S3; ^{13}C NMR (DMSO-d_6/$CDCl_3$) see Table S4; HPLC–MS (general gradient) R_t = 13.8 min; MS^2 395.2, 367.7, 356.2, 348.6, 312.3, 296.2, 284.7, 268.1, 255.2, 244.9, 231.0, 216.9, 174.9, 158.9, 131.1; (+)-HRMS 413.3163 *m/z* [M + H]$^+$ (calcd for $C_{26}H_{40}N_2O_2$ *m/z* [M + H]$^+$ 413.3163).

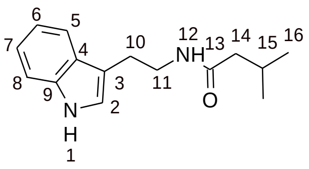

20

Compound **20**, *N*-[2-(1*H*-indol-3-yl)ethyl]-3-methylbutanamide: white solid; ^{1}H NMR (DMSO-d_6) see Table S5; ^{13}C NMR (DMSO-d_6) see Table S6; HPLC–MS (general gradient) R_t = 7.6 min; MS^2 228.0, 161.0, 144.0; (+)-HRMS 245.1654 *m*/*z* [M + H]$^+$ (calcd for $C_{15}H_{20}N_2O$ *m*/*z* [M + H]$^+$ 245.1648).

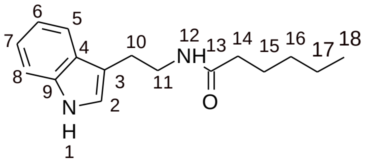

21

Compound **21**, *N*-[2-(1*H*-indol-3-yl)ethyl]hexanamide: white solid; ^{1}H NMR (DMSO-d_6) see Table S5; ^{13}C NMR (DMSO-d_6) see Table S6; HPLC–MS (general gradient) R_t = 8.5 min; MS^2 176.8, 161.0, 144.0; (+)-HRMS 259.1809 *m*/*z* [M + H]$^+$ (calcd for $C_{16}H_{22}N_2O$ *m*/*z* [M + H]$^+$ 259.1805).

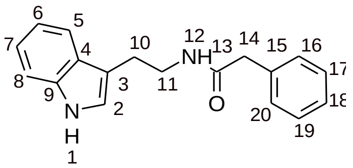

22

Compound **22**, *N*-[2-(1*H*-indol-3-yl)ethyl]-2-phenylacetamide: white solid; ^{1}H NMR (DMSO-d_6) see Table S5; ^{13}C NMR (DMSO-d_6) see Table S6; HPLC–MS (general gradient) R_t = 8.1 min; MS^2 262, 161, 144; (+)-HRMS 279.1496 *m*/*z* [M + H]$^+$ (calcd for $C_{18}H_{18}N_2O$ *m*/*z* [M + H]$^+$ 279.1492).

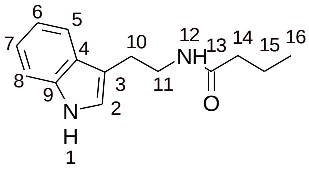

23

Compound **23**, *N*-[2-(1*H*-indol-3-yl)ethyl]butanamide: white solid; ^{1}H NMR (DMSO-d_6) see Table S5; ^{13}C NMR (DMSO-d_6) see Table S6; HPLC–MS (general gradient) R_t = 7.1 min; MS^2 214.0, 161.0, 144.0; (+)-HRMS 231.1483 *m*/*z* [M + H]$^+$ (calcd for $C_{14}H_{18}N_2O$ *m*/*z* [M + H]$^+$ 231.1492).

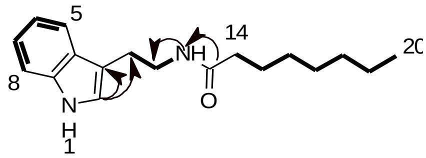

24

Compound **24**, *N*-[2-(1*H*-indol-3-yl)ethyl]octanamide: white solid; UV (DMSO) λ_{max} (ε) 284.0 (4200); IR ν_{max}/cm^{-1} 3397, 3252, 3080, 2916, 2849, 1651, 1630, 1566, 1456, 1425, 1374, 1094; ^{1}H NMR (DMSO-d_6) see Table S5; ^{13}C NMR (DMSO-d_6) see Table S6; HPLC–MS (general gradient) R_t = 9.8 min; MS^2 270.1, 161.0, 144.0; (+)-HRMS 287.2120 *m*/*z* [M + H]$^+$ (calcd for $C_{18}H_{26}N_2O$ *m*/*z* [M + H]$^+$ 287.2118).

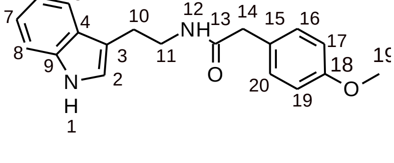

25

Compound **25**, *N*-[2-(1*H*-indol-3-yl)ethyl]-2-(4-methoxyphenyl)acetamide: white solid; ^{1}H NMR (DMSO-d_6) see Table S5; ^{13}C NMR (DMSO-d_6) see Table S6; HPLC–MS

(general gradient) R_t = 8.1 min; MS^2 292.0, 161.0, 144.0; (+)-HRMS 309.1598 *m*/*z* $[M + H]^+$ (calcd for $C_{19}H_{20}N_2O_2$ *m*/*z* $[M + H]^+$ 309.1598).

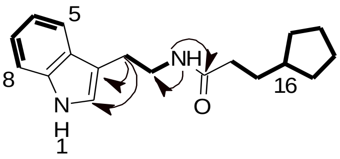

26

Compound **26**, 3-cyclopentyl-*N*-[2-(1*H*-indol-3-yl)ethyl]propanamide: yellowish oil; UV (DMSO) λ_{max} (ε) 284.0 (5700); IR ν_{max}/cm^{-1} 3289, 3080, 2940, 2857, 1619, 1555, 1455, 1353, 1229, 1103; ^{1}H NMR (DMSO-d_6) see Table S5; ^{13}C NMR (DMSO-d_6) see Table S6; HPLC–MS (general gradient) R_t = 9.1 min; MS^2 268.0, 161.0, 144.0; (+)-HRMS 285.1962 *m*/*z* $[M + H]^+$ (calcd for $C_{18}H_{24}N_2O$ *m*/*z* $[M + H]^+$ 285.1961).

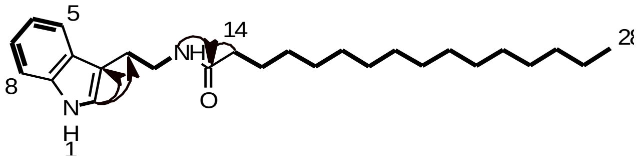

27

Compound **27**, *N*-[2-(1*H*-indol-3-yl)ethyl]hexadecanamide: white solid; UV (DMSO) λ_{max} (ε) 284.0 (5400); IR ν_{max}/cm^{-1} 3392, 3268, 3086, 2918, 2849, 1649, 1630, 1556, 1455, 1209, 1094; ^{1}H NMR (DMSO-d_6) see Table S5; ^{13}C NMR (DMSO-d_6) see Table S6; HPLC–MS (general gradient) R_t = 14.1 min; MS^2 382.1, 356.6, 311.2, 299.6, 281.1, 268.9, 230.9, 161.0, 144.0; (+)-HRMS 399.3376 *m*/*z* $[M + H]^+$ (calcd for $C_{26}H_{42}N_2O$ *m*/*z* $[M + H]^+$ 399.3370)

Table S1: ^{1}H NMR of compounds **3–11**.

Position	**3**	**4**	**5**	**6**	**8**	**9**	**10**	**11**
1	8.57 (bs, 1H)	8.31 (bs, 1H)	8.32 (bs, 1H)	8.53 (bs, 1H)	8.60 (bs, 1H)	8.47 (bs, 1H)	8.42 (bs, 1H)	8.40 (bs, 1H)
2	7.49 (d, 2.5, 1H)	7.49 (d, 2.5,1H)	7.47 (d, 2.5, 1H)	7.52 (d, 2.5, 1H)	7.52 (d, 2.5, 1H)	7.48 (s, 1H)	7.52 (d, 2.4, 1H)	7.52 (d, 2.3, 1H)
3								
4								
5	7.41 (d, 7.0, 1H)	7.41 (d, 7.5, 1H)	7.40 (d, 7.5, 1H)	7.44 (d, 7.7, 1H)	7.44 (d, 7.8, 1H)	7.42 (d, 8.6, 1H)	7.44 (d, 7.5, 1H)	7.44 (d, 7.9, 1H)
6	7.21 (dd, 7.5, 1H)	7.21 (dd, 7.0, 1H)	7.26 (m)	7.27 (m, 2H)	7.27 (m, 2H)	7.36 (m, 1H)	7.27 (m, 2H)	7.27 (m, 2H)
7	7.27 (dd, 7.5, 1H)	7.27 (dd, 7.0, 1H)	7.26 (m)	7.27 (m, 2H)	7.27 (m, 2H)	7.36 (m, 1H)	7.27 (m, 2H)	7.27 (m, 2H)
8	7.83 (d, 7.5, 1H)	7.82 (d, 7.5, 1H)	7.78 (d, 7.5, 1H)	7.85 (d, 7.7, 1H)	7.86 (d, 7.6, 1H)	7.81 (d, 7.8, 1H)	7.85 (d, 7.6, 1H)	7.85 (d, 7.7, 1H)
9								
10								
11	7.16 (s, 1H)	7.13 (s, 1H)	7.17 (s, 1H)	7.17 (s, 1H)	7.17 (s, 1H)	7.18 (s, 1H)	7.16 (s, 1H)	7.16 (s, 1H)
12								
13								
14	2.71 (d, 7.0, 2H)	2.83 (t, 7.5, 2H)	4.19 (s, 2H)	2.84 (t, 7.5, 2H)	2.86 (t, 7.6, 2H)	4.14 (s, 2H)	2.87 (t, 7.5, 2H)	2.85 (t, 7.6, 2H)
15	2.22 (m, 1H)	1.82 (m, 2H)		1.89 (m, 2H)	1.86 (q, 7.5, 2H)		1.85 (m, 5H)	1.85 (q, 7.5, 2H)
16	1.02 (d, 6.5, 6H)	1.39 (m, 2H)	7.34 (m, 2H)	1.06 (t, 7.4, 3H)	1.37 (m, 8H)	7.36 (m, 2H)	1.85 (m, 5H)	1.35 (m, 22H)
17		1.37 (m, 2H)	7.34 (m, 2H)		1.37 (m, 8H)	6.89 (m, 2H)	1.18 (m, 2H), 1.85 (m, 5H)	1.35 (m, 22H)
18		0.90 (t, 7.0, 3H)	7.24 (m, 1H)		1.37 (m, 8H)		1.60 (m, 4H)	1.35 (m, 22H)
19			7.34 (m, 2H)		1.37 (m, 8H)	7.36 (m, 2H)	1.60 (m, 4H)	1.35 (m, 22H)
20			7.34 (m, 2H)		0.89 (t, 6.8, 3H)	6.89 (m, 2H)	1.18 (m, 2H), 1.85 (m, 5H)	1.35 (m, 22H)
21						3.06 (s, 3H)		1.35 (m, 22H)
22								1.35 (m, 22H)
23								1.35 (m, 22H)
24								1.35 (m, 22H)
25								1.35 (m, 22H)
26								1.35 (m, 22H)
27								1.35 (m, 22H)
28								0.89 (t, 6.8, 3H)

Table S2: ^{13}C NMR of compounds **3–11**.

Position	**3**	**4**	**5**	**6**	**8**	**9**	**10**	**11**
1								
2	121.5	121.4	121.7	121.5	121.5	121.7	121.5	121.4
3	105.9	106.2	106	106.1	106	105.8	106.1	106.2
4	124	124.1	124.1	124.1	124.1	124.1	124.1	124.1
5	111.5	111.5	111.5	111.5	111.5	111.5	111.5	111.5
6	122.9	123	123.1	123	123	123	123	123
7	120.8	120.9	120.9	120.8	120.8	120.8	120.8	120.8
8	119.9	120	120	120	120	119.9	120	120
9	136.2	136	136.2	136.2	136.2	136.2	136.2	136.2
10	147.2	147	147.8	147.1	147.1	147.7	147.1	147
11	119.7	119.8	119.9	119.8	119.6	120	119.7	119.9
12								
13	162.3	163	160.9	162	163	161.2	163.1	163
14	37.1	28.2	34.8	30.1	28.2	33.9	27.5	28.2
15	27.7	26.9	135.9	20.6	27.2	127.8	33.4	27.1
16	22.4 (2C)	31.4	128.7	13.77	29.2	129.9	39.7	29.2*
17		22.3	128.8		28.9	114.1	32.5	29.3*
18		14	127		31.7	158.6	25.2	29.4*
19			128.7		22.6	129.9	25.2	29.5*
20			128.8		14.1	114.1	32.5	29.6*
21						55.3		29.7*
22								29.7*
23								29.7*
24								29.7*
25								29.7*
26								31.9*
27								22.7
28								14.1

Table S3: ^{1}H NMR of compounds **12–19**.

Position	**12**	**13**	**14**	**15**	**16**	**17**	**18**	**19**
1	11.98 (bs, 1H)	11.98 (bs, 1H)	12.00 (bs, 1H)	12.00 (bs, 1H)	11.98 (bs, 1H)	10.80 (bs, 1H)	11.98 (bs, 1H)	10.73 (bs, 1H)
2	8.42 (d, 3.1, 1H)	8.39 (s, 1H)	8.41 (s, 1H)	8.40 (s, 1H)	8.41 (d, 3.0, 1H)	8.40 (s, 1H)	8.41 (d, 3.1, 1H)	8.26 (d, 3.1, 1H)
3								
4								
5	7.48 (d, 6.8, 1H)	7.46 (m, 1H)	7.48 (d, 7.3, 1H)	7.46 (m, 1H)	7.48 (d, 6.7, 1H)	7.48 (d, 7.0, 1H)	7.48 (m, 1H)	8.17 (d, 7.0, 1H)
6	7.21 (m, 7.1, 2H)	7.19 (m, 2H)	7.21 (m, 3H)	7.19 (m, 2H)	7.21 (m, 7.0, 1H)	7.24 (m, 4H)	7.21 (m, 2H)	7.17 (m, 2H)
7	7.21 (m, 7.1, 2H)	7.19 (m, 2H)	7.21 (m, 3H)	7.19 (m, 2H)	7.21 (m, 7.0, 1H)	7.24 (m, 4H)	7.21 (m, 2H)	7.17 (m, 2H)
8	8.17 (d, 6.9, 1H)	8.11 (m, 2H)	8.17 (d, 7.3, 1H)	8.15 (m, 1H)	8.16 (d, 6.7, 1H)	8.16 (d, 7.0, 1H)	8.16 (m,1H)	7.44 (d, 7.9, 1H)
9								
10								
11	4.46 (d, 5.7, 2H)	4.43 (d, 5.6, 2H)	4.48 (d, 5.4, 2H)	4.43 (d, 5.7, 2H)	4.45 (d, 5.7, 2H)	4.48 (d, 5.5, 2H)	4.45 (d, 5.7, 2H)	4.46 (d, 5.4, 2H)
12	8.09 (t, 5.6, 1H)	8.11 (m, 2H)	8.34 (t, 5.3, 1H)	8.08 (m, 1H)	8.09 (t, 5.6, 1H)	8.25 (t, 5.5, 1H)	8.10 (t, 5.6, 1H)	7.94 (t, 5.4, 1H)
13								
14	2.05 (m, 3H)	2.17 (t, 7.4, 2H)	3.32 (s, 2H)	2.16 (t, 7.3, 2H)	2.19 (t, 7.4, 2H)	3.48 (s, 2H)	2.21 (t, 7.5, 2H)	2.20 (t, 7.5, 2H)
15	2.05 (m, 3H)	1.52 (m, 2H)		1.54 (m, 2H)	1.53 (m, 2H)		1.5 (m, 4H)	1.56 (m, 2H)
16	0.93 (d, 6.4, 6H)	1.26 (m, 4H)	7.31 (m, 4H)	0.88 (t, 7.4, 3H)	1.27 (m, 8H)	7.24 (m, 4H)	1.74	1.23 (m, 24H)
17		1.26 (m, 4H)	7.31 (m, 4H)		1.27 (m, 8H)	6.88 (m, 2H)	1.09 (m, 2H), 1.74 (m, 3H)	1.23 (m, 24H)
18		0.85 (t, 6.7, 3H)	7.21 (m, 3H)		1.27 (m, 8H)		1.5 (m, 6H)	1.23 (m, 24H)
19			7.31 (m, 4H)		1.27 (m, 8H)	6.88 (m, 2H)	1.5 (m, 6H)	1.23 (m, 24H)
20			7.31 (m, 4H)		0.87 (m, 3H)	7.24 (m, 4H)	1.09 (m, 2H), 1.74 (m, 3H)	1.23 (m, 24H)
21						3.74 (s, 3H)		1.23 (m, 24H)
22								1.23 (m, 24H)
23								1.23 (m, 24H)
24								1.23 (m, 24H)
25								1.23 (m, 24H)
26								1.23 (m, 24H)
27								1.23 (m, 24H)
28								0.85 (t, 6.8, 3H)

Table S4: ^{13}C NMR of compounds **12–19**.

Position	**12**	**13**	**14**	**15**	**16**	**17**	**18**	**19**
1								
2	133.5	133.5	133.6	133.5	133.4	133.6	133.5	132.8
3	114.1	114.0	114.0	114.0	114.0	114.0	114.1	114.0
4	125.4	125.3	125.3	125.3	125.4	124.4	125.4	125.3
5	112.1	112.1	112.1	112.1	112.1	112.2	112.1	121.1
6	121.8	121.8	121.8	121.8	121.7	121.8	122.8	122.6
7	122.8	122.8	122.8	122.8	122.8	122.9	121.8	121.6
8	121.1	121.1	121.1	121.1	121.1	121.1	121.1	111.9
9	136.4	136.4	136.4	136.4	136.4	136.4	136.4	136.4
10	190.4	190.4	190.1	190.4	190.4	190.2	190.4	190.0
11	45.6	45.6	45.8	45.6	45.6	45.8	45.6	45.6
12								
13	171.8	172.4	170.3	172.3	172.4	170.7	172.5	172.6
14	44.6	35.2	42.1	37.1	35.2	41.3	34.6	35.4
15	25.6	25.0	125.3	18.7	25.3	128.3	31.6	25.3
16	22.4 (2C)	30.9	128.1*	13.6	28.6	130.1	39.2	28.7*
17		21.9	129.0		28.5	113.6	32.0	28.8*
18		13.9	126.3*		31.2	157.9	24.7	28.9*
19			129.0		22.0	113.6	24.7	29.0*
20			128.1*		13.9	130.1	32.0	29.0*
21						55.0		29.1*
22								29.1*
23								29.1*
24								29.1*
25								29.1*
26								31.3*
27								22.1
28								13.8

Table S5: ^{1}H NMR of compounds **20–27**.

Position	**20**	**21**	**22**	**23**	**24**	**25**	**26**	**27**
1	10.27 (bs, 1H)	10.79 (bs, 1H)	10.90 (bs, 1H)	10.79 (bs, 1H)	10.75 (bs, 1H)	10.80 (bs, 1H)	10.79 (bs, 1H)	10.73 (bs, 1H)
2	7.11 (d, 2.2, 1H)	7.13 (d, 2.1, 1H)	7.11 (s, 1H)	7.14 (s, 1H)	7.07 (s, 1H)	7.10 (s, 1H)	7.13 (s, 1H)	7.07 (d, 2.0, 1H)
3								
4								
5	7.31 (d, 8.0, 1H)	7.33 (d, 8.1, 1H)	7.28 (m, 6H)	7.33 (d, 8.1, 1H)	7.27 (d, 8.0, 1H)	7.34 (m, 1H)	7.34 (d, 8.1, 1H)	7.46 (d, 7.8, 1H)
6	6.96 (m, 1H)	6.98 (m, 1H)	6.97 (m, 1H)	6.98 (m, 1H)	6.91 (m, 1H)	6.98 (m, 1H)	6.98 (m, 1H)	6.91 (m, 1H)
7	7.04 (m, 1H)	7.06 (m, 1H)	7.07 (M, 1H)	7.06 (m, 1H)	7.00 (m, 1H)	7.07 (m, 1H)	7.06 (m, 1H)	7.00 (m, 1H)
8	7.51 (d, 7.9, 1H)	7.53 (d, 7.8, 1H)	7.53 (d, 7.8, 1H)	7.53 (d, 7.8, 1H)	7.46 (d, 7.8, 1H)	7.52 (d, 7.9, 1H)	7.53 (d, 7.9, 1H)	7.27 (d, 8.1, 1H)
9								
10	2.79 (t, 7.4, 2H)	2.81 (t, 7.4, 2H)	2.83 (t, 7.4, 2H)	2.81 (t, 7.4, 2H)	2.74 (t, 7.4, 2H)	2.82 (t, 7.4, 2H)	2.81 (t, 7.4, 2H)	2.74 (t, 7.4, 2H)
11	3.31 (t, 7.4, 2H)	3.31 (t, 7.4, 2H)	3.33 (m, 4H)	3.33 (m, 2H)	3.27 (m, 2H)	3.33 (m, 4H)	3.32 (t, 7.4, 2H)	3.25 (t, 7.5, 2H)
12	7.85 (t, 5.5, 1H)	7.86 (t, 5.5, 1H)	8.13 (t, 5.5, 1H)	7.86 (t, 5.4, 1H)	7.82 (t, 5.5, 1H)	8.04 (t, 5.5, 1H)	7.87 (t, 5.5, 1H)	7.80 (t, 5.6, 1H)
13								
14	1.93 (m, 3H)	2.03 (m, 2H)	3.33 (m, 4H)	2.04 (t, 7.3, 2H)	1.99 (t, 7.4, 2H)	3.33 (m, 4H)	2.07 (t, 7.5, 2H)	1.99 (t, 7.4, 2H)
15	1.93 (m, 3H)	1.49 (m, 2H)	7.28 (m, 6H)	1.52 (m, 2H)	1.43 (m, 2H)	7.16 (m, 2H)	1.51 (m, 2H)	1.42 (q, 6.8, 2H)
16	0.84 (m, 6H)	1.24 (m, 4H)	7.28 (m, 6H)	0.85 (t, 7.5, 3H)	1.18 (m, 8H)	6.85 (m, 2H)	1.70 (m, 3H)	1.18 (bs, 24H)
17	0.84 (m, 6H)	1.24 (m, 4H)	7.28 (m, 6H)		1.18 (m, 8H)		1.04 (m, 2H), 1.70 (m, 3H)	1.18 (bs, 24H)
18		0.86 (m, 3H)	7.28 (m, 6H)		1.18 (m, 8H)	6.85 (m, 2H)	1.51 (m, 4H)	1.18 (bs, 24H)
19			7.28 (m, 6H)		1.18 (m, 8H)	7.16 (m, 2H)	1.51 (m, 4H)	1.18 (bs, 24H)
20					0.80 (t, 6.9, 3H)	3.73 (s, 3H)	1.04 (m, 2H), 1.70 (m, 3H)	1.18 (bs, 24H)
21								1.18 (bs, 24H)
22								1.18 (bs, 24H)
23								1.18 (bs, 24H)
24								1.18 (bs, 24H)
25								1.18 (bs, 24H)
26								1.18 (bs, 24H)
27								1.18 (bs, 24H)
28								0.80 (t, 7.0, 3H)

Table S6: ^{13}C NMR of compounds **20–27**.

Position	**20**	**21**	**22**	**23**	**24**	**25**	**26**	**27**
1								
2	122.5	122.5	122.6	122.5	122.5	122.6	122.6	122.5
3	111.8	111.9	111.7	111.9	111.8	111.7	111.9	111.8
4	127.2	127.2	127.2	127.2	127.2	127.2	127.3	127.2
5	111.3	111.3	111.3	111.3	111.3	111.3	111.3	111.3
6	118.2	118.1	118.2	118.2	118.1	118.2	118.2	118.2
7	120.9	120.8	120.8	120.9	120.8	120.8	120.9	120.8
8	118.2	118.2	118.2	118.3	118.2	118.2	118.2	118.2
9	136.2	136.2	136.2	136.2	136.2	136.2	136.2	136.2
10	25.5	25.2	25.1	25.3	25.2	25.2	25.2	25.6
11	39.3	39.4	39.6	39.4	39.3	39.6	39.4	39.4
12								
13	171.3	171.9	169.9	171.8	171.9	170.3	172.1	171.9
14	44.8	35.4	42.4	37.4	35.4	41.5	34.8	35.4
15	25.3	24.9	136.5	18.7	25.3	129.9	31.6	25.2
16	22.3	30.9	128.9	13.7	28.6	113.4	39.2	28.6*
17	22.3	21.9	128.1		28.4	157.8	32.0	28.7*
18		13.8	126.2		31.1	113.4	24.7	28.8*
19			128.1		22.0	129.9	24.7	28.9*
20			128.9		13.9	55.0	32.0	29.0*
21								29.0*
22								29.0*
23								29.0*
24								29.0*
25								29.0*
26								31.2*
27								22.0
28								13.9

*****Interchangeable position.

Table S7: Bioactivity results (- = not active; * = crystallization at concentrations required for the assay). Compounds **12**–**19** were not active in the MCF-7 cell assay and crystallized at concentrations required for the *Galleria* assay.

Compound	*Galleria* hemocytes LD_{50} (µg ml^{-1})	MCF-7 EC_{50} (µg ml^{-1})
**3**	29.76	58
**4**	*	—
**5**	*	363
**6**	1.65	25.7
**7**	—	—
**8**	—	—
**9**	103.1*	34.5
**10**	*	—
**11**	*	—
**20**	92.99	—
**21**	1.27	—
**22**	22.44	—
**23**	48.76	—
**24**	1.02	—
**25**	*	—
**26**	2.43	111.5
**27**	*	—

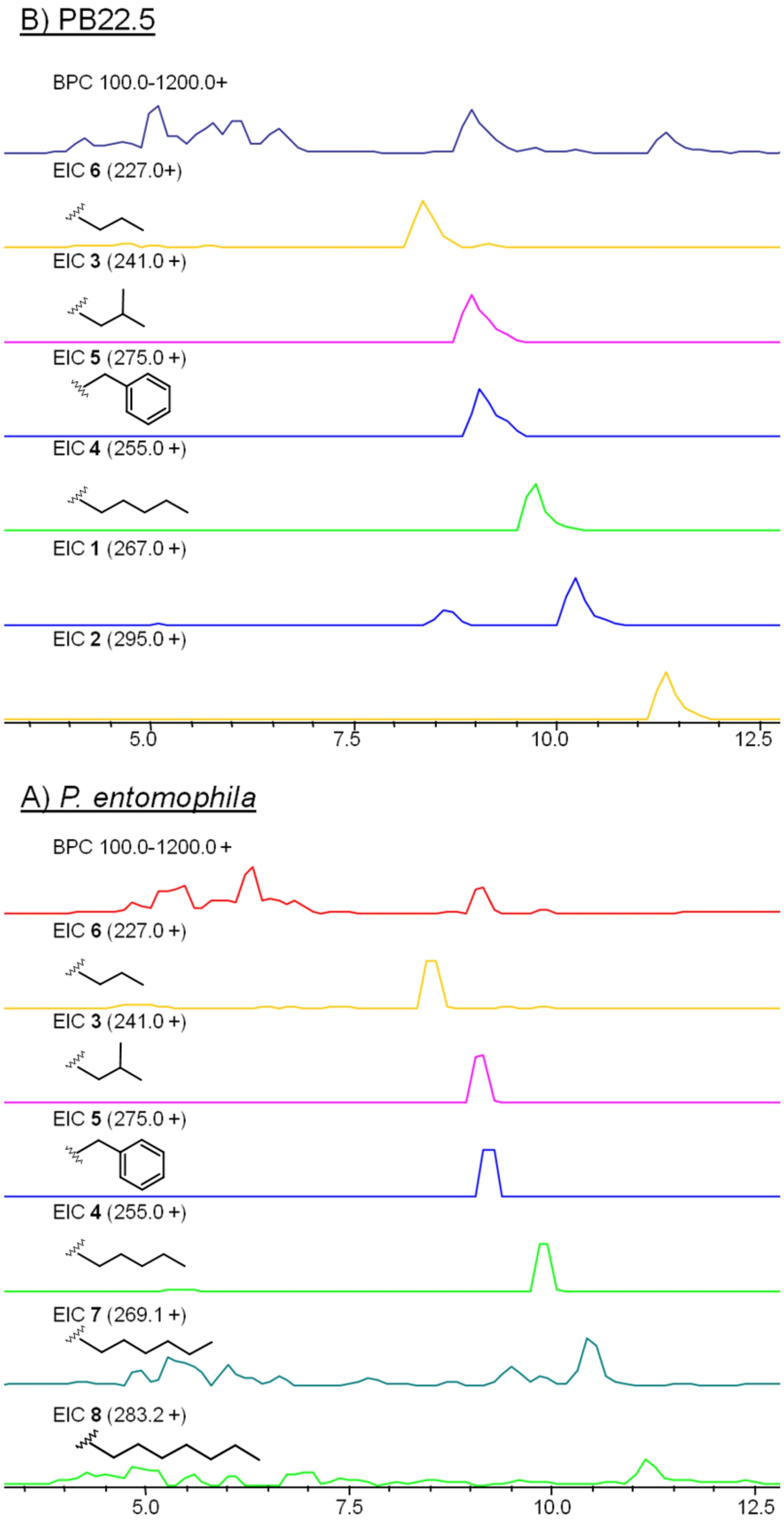

Figure S1: A) HPLC–MS base-peak chromatograms of the crude extract and extracted ion chromatograms of the natural compounds of *P.entomophila* (**A**) and PB22.5 (**B**). The different moieties of the oxazoles are shown above the chromatogram.

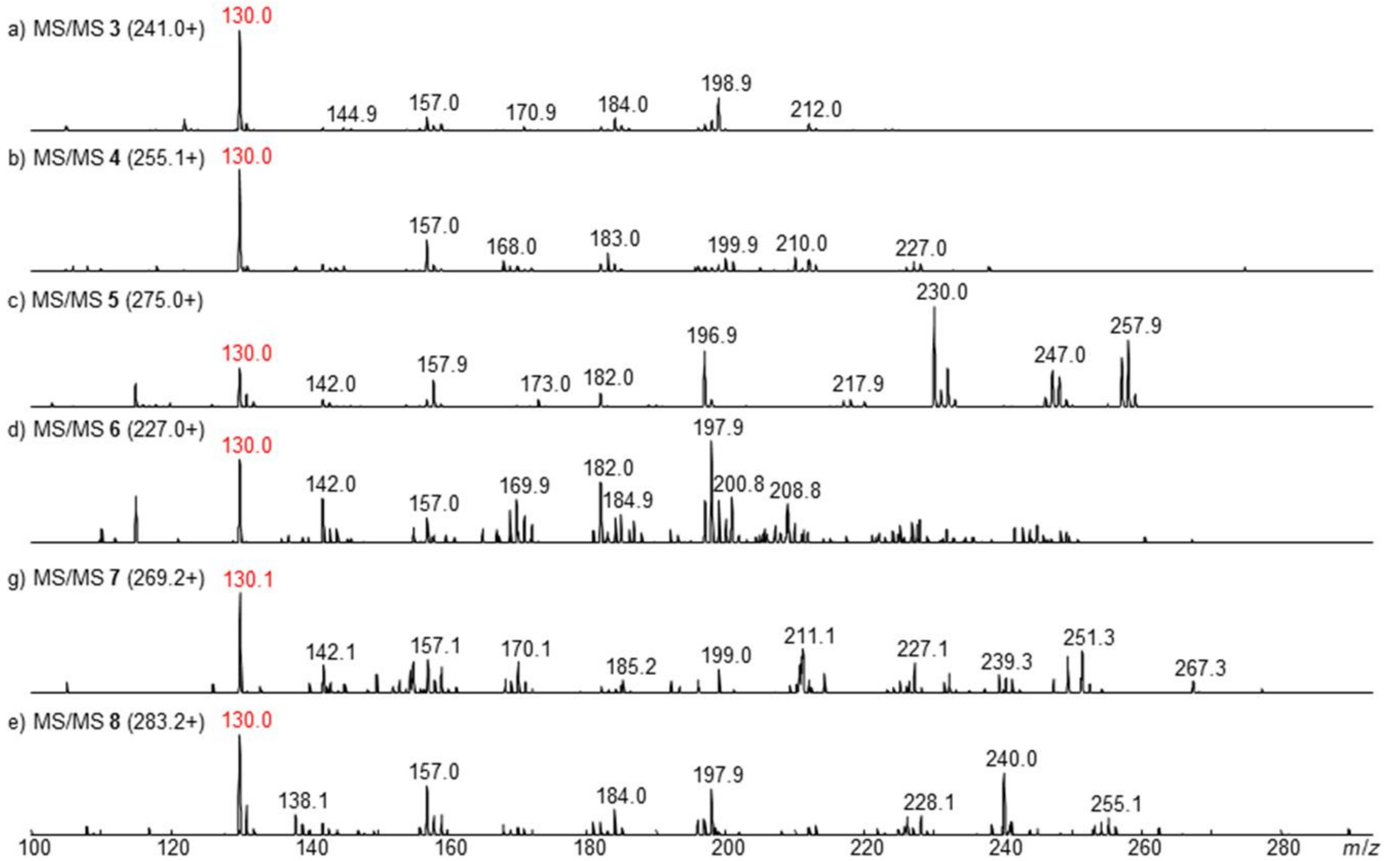

Figure S2: MS/MS spectra of the synthetic products **3**–**8** and the identified natural oxazoles **7** (g) and **8** (f) from *P. entomophila.* The characteristic oxazole fragment is shown in red.

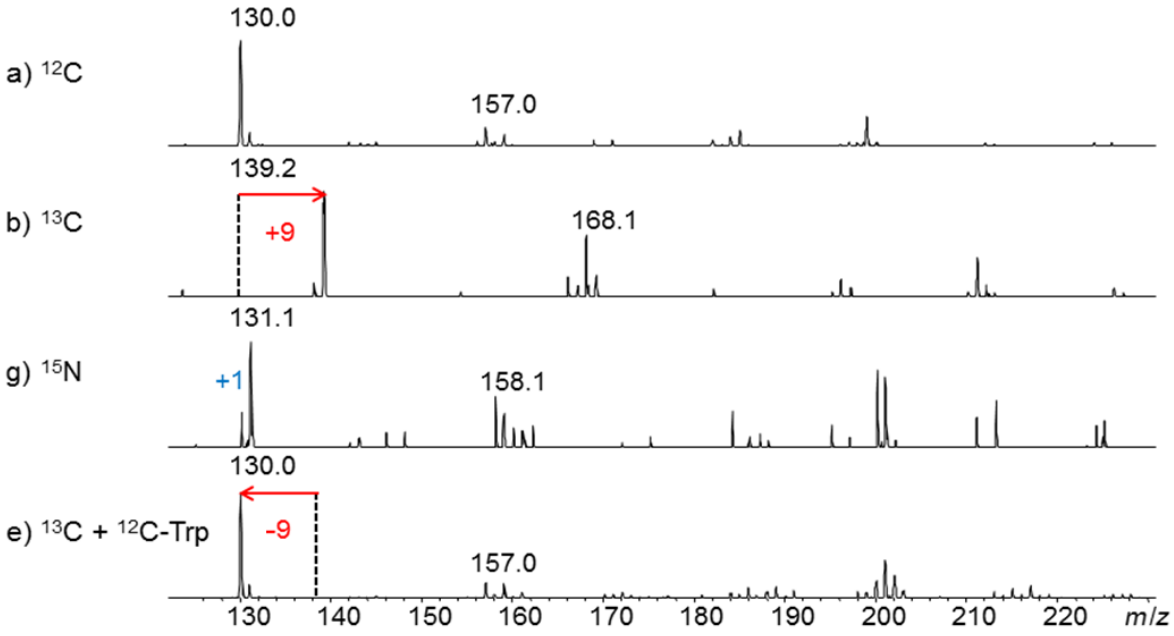

Figure S3: MS/MS spectra of compound **3** with isotopic labeling as proof of the structure of the characteristic fragment m/z = 130.

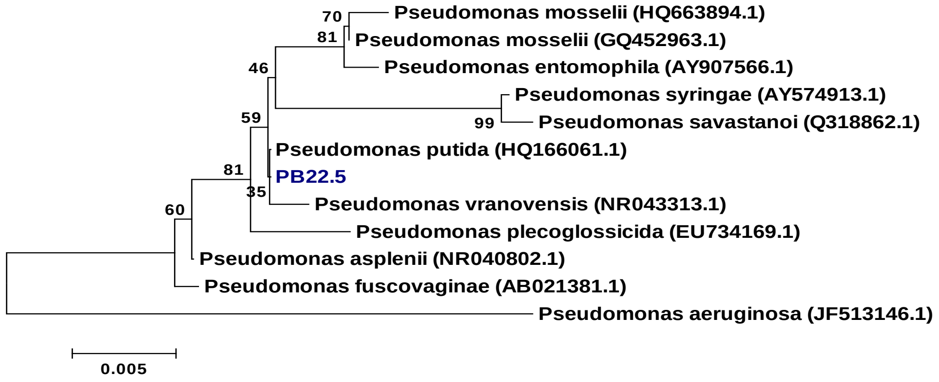

Figure S4: Phylogenetic relationships of PB22.5 and *Pseudomonas* sp. The tree was constructed by using the partial 16S rDNA gene sequence (537 bp), the model of Kimura 2-parameter and the Neighbor-Joining module of MEGA software version 4.0.2. A bootstrap value is 1,000 replicates. The bar indicates 0.5% sequence divergence. Numbers after each species indicate the GenBank Accession numbers**.**

References

1. Akhurst, R. J. *J. Gen. Microbiol.* **1980**, *121*, 303–309.
2. White, G. F. *Science* **1927**, *66*, 302–303.
3. Woodring, L. J. H.; Kaya, K. H. *Steinernematid and Heterorhabditid nematodes. A Handbook of biology and techniques*; Southern Cooperative Series Bulletin, Arkansas Agricultural Experimental Station: Fayetteville Arkansas, USA, **1988**.
4. Thompson, J. D.; Higgins, D. G.; Gibson, T. J. *Nucleic Acids Res.* **1994**, *22*, 4673–4680.
5. Tamura, K.; Dudley, J.; Nei, M.; Kumar, S. *Mol. Biol. Evol.* **2007**, *24*, 1596–1599.
6. Saitou, N.; Nei, M. *Mol. Biol. Evol.* **1987**, *4*, 406–425.
7. Pettit, G. R.; Knight, J. C.; Herald, D. L.; Davenport, R.; Pettit, R. K.; Tucker, B. E.; Schmidt, J. M. *J. Nat. Prod.* **2002**, *65*, 1793–1797.
